# Continuous-Wave Laser-Induced Transfer of Metal Nanoparticles to Arbitrary Polymer Substrates

**DOI:** 10.3390/nano10040701

**Published:** 2020-04-07

**Authors:** Jaemook Lim, Youngchan Kim, Jaeho Shin, Younggeun Lee, Wooseop Shin, Weihao Qu, Eunseung Hwang, Seongje Park, Sukjoon Hong

**Affiliations:** 1 Optical Nanoprocessing Lab, Department of Mechanical Engineering, Hanyang University, Ansan 15588, Korea; limjaemook@hanyang.ac.kr (J.L.); geows3@hanyang.ac.kr (Y.K.); twilit77@hanyang.ac.kr (Y.L.); qwh80802@gmail.com (W.Q.); joseph5017@hanyang.ac.kr (E.H.); seongje@kitech.re.kr (S.P.); 2 Applied Nano and Thermal Science Lab, Department of Mechanical Engineering, Seoul National University, Seoul 08826, Korea; jayz.shin84@gmail.com (J.S.); slf920923@gmail.com (W.S.)

**Keywords:** laser-induced forward transfer, selective laser sintering, metal nanoparticle ink, flexible electronics

## Abstract

Laser-induced forward transfer (LIFT) and selective laser sintering (SLS) are two distinct laser processes that can be applied to metal nanoparticle (NP) ink for the fabrication of a conductive layer on various substrates. A pulsed laser and a continuous-wave (CW) laser are utilized respectively in the conventional LIFT and SLS processes; however, in this study, CW laser-induced transfer of the metal NP is proposed to achieve simultaneous sintering and transfer of the metal NP to a wide range of polymer substrates. At the optimum laser parameters, it was shown that a high-quality uniform metal conductor was created on the acceptor substrate while the metal NP was sharply detached from the donor substrate, and we anticipate that such an asymmetric transfer phenomenon is related to the difference in the adhesion strengths. The resultant metal electrode exhibits a low resistivity that is comparable to its bulk counterpart, together with strong adhesion to the target polymer substrate. The versatility of the proposed process in terms of the target substrate and applicable metal NPs brightens its prospects as a facile manufacturing scheme for flexible electronics.

## 1. Introduction

Wearable electronics such as skin-attachable medical devices may have a huge impact on our daily lives [1]. As a consequence, the development of adequate manufacturing methods to produce flexible electronics, which are one of the basic elements for a wearable device, is becoming increasingly important [2,3] as well. As potential constituent materials for these flexible electronics, nanomaterials such as nanotubes, nanowires, and nanofibers are under active investigation [4,5], whilst nanoparticles (NPs) are drawing keen attention, since a conductive layer can be created on various substrates through a simple sintering process at a relatively low temperature [6,7].

Selective laser sintering (SLS) was recently in the spotlight as a sintering scheme for these metal NPs. The direct writing nature of SLS enables the simultaneous patterning and sintering of metal NPs, which is advantageous for the rapid maskless adjustment of the electrode design. Moreover, since the laser parameters are highly controllable, the SLS process was confirmed to be applicable to the creation of a metal layer on flexible substrates which are sensitive to heat. The SLS process was first applied to noble metal NPs such as gold (Au) [8] and silver (Ag) [9,10], but the range of applicable NPs is being expanded to include more cost-effective materials such as copper (Cu) [11,12] and nickel (Ni) [13,14] through the reductive sintering process.

In the conventional SLS process for metal NPs, a focused laser is utilized as a localized heat source to sinter metal NPs selectively along the designated path. While the deposition of metal NPs on the target substrate is a prerequisite for the SLS process [10,15], the uniform coating of the metal NP ink is often problematic for certain flexible films due to its hardness in handling and the poor wettability between the substrate and the NP ink under concern [16]. The additional cleaning process after the SLS process, which is required to remove the remaining NP ink from the substrate [9,12], also remains as a redundant step.

To overcome these limitations, non-contact methods for metal NP patterning such as LIFT process have been investigated. The LIFT process utilizes a pulsed laser beam as a driving force to transfer the material from the donor substrate to the acceptor substrate, which are in proximity [17,18]. The NPs transferred by a single pulse have a circular shape in general [19], and therefore, the single transfer has to be repeated at specific laser parameters and a proper hatch size to integrate each droplet and create continuous features [20,21]. Furthermore, unlike the SLS process, additional heat treatment is essential for these metal NPs to function properly as electrodes [20,22]. Since the LIFT process and the SLS process use a pulsed laser and a continuous-wave (CW) laser, respectively, an additional furnace or another optical system is required for the heat treatment after the transfer step, which inevitably increases the overall manufacturing cost.

Some techniques that performed the SLS process with pulse laser or LIFT process with CW laser have been studied to overcome these innate limitations. P. Sopeña et al. proposed the CW-LIFT method [23] and analyzed the dynamics of the method [24], which uses a CW laser for the LIFT of the liquid Ag NP ink, and F. Zacharatos et al. [25] introduced a sintering method using a high-repetition-rate pulsed laser after the conventional LIFT method. However, both of these methods could not achieve the simultaneous sintering and transfer of the target material.

In this report, we introduce the CW laser-induced transfer of metal NP ink to tackle the preceding problems. The transfer mechanism of the conventional LIFT process is to create impulsive heating at the donor substrate by a laser pulse, to create a jet from the donor to the acceptor that often suffers from high irreproducibility [26]. In this study, a pulsed laser is substituted with a CW laser, and the laser transfer is achieved by harnessing the difference in the adhesion strengths between the sintered metal NPs and the donor/acceptor substrates. Since sintering and transfer occur simultaneously with the CW laser-induced photothermal reaction, a high-quality continuous conductive structure is created without any post-cleaning or additional heat treatment, which has been compulsory in the conventional SLS and LIFT processes. The potential of the proposed method is then further verified by its successful application on various polymer substrates, including commercial products.

## 2. Materials and Methods

### 2.1. Material Preparation and CW Laser-Induced Metal NP Transfer Method

**Figure 1:**
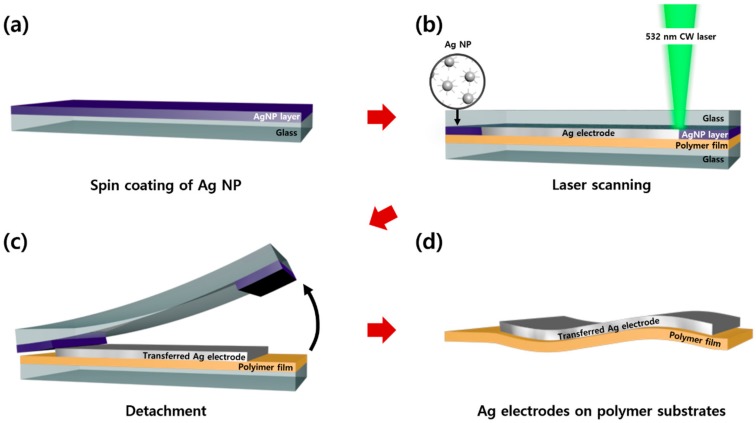
Schematics of the CW laser-induced Ag NP transfer to the thin polymer film. (**a**) Spin coating of the Ag NP ink onto the donor glass substrate. (**b**) Laser scanning conducted at the Ag NP layer, which is sandwiched between the glass substrate and the polymer film. (**c**) The detachment of the donor substrate with the Ag NP. (**d**) Ag electrode on the acceptor polymer film transferred from the donor substrate.

For the development of the the CW laser-induced metal NP transfer method and comparison of the results with the previous studies, Ag NP ink was selected as the representative metal NP ink [15,27]. The current study was conducted with commercial Ag NP ink (NPS-J, Harima Chemicals, Inc. Tokyo, Japan) and used without any purification after the purchase. The overall experimental steps are schematically illustrated in Figure 1. In a typical experiment, Ag NP ink was first coated onto the donor glass substrate (microscope slides of 1 mm thickness, Marienfeld, Lauda-Königshofen, Baden-Württemberg, Germany) using a spin coater (ACE-200, Dong Ah Trade Corp., Seoul, Korea) at 300 rpm for 200 s (Figure 1a). To control the wettability of the coated Ag NP, the donor glass substrate was dried on a hot plate (MSH-30D, Daihan Scientific, Wonju, Korea) at 60 °C for 20 min. After the spin coating process, the Ag NP layer on the donor glass substrate was flipped over to make contact with a polymer film which was placed on a glass substrate. As the Ag NP was almost dried, it was not spontaneously transferred when it touched the polymer film [28]. This process was confirmed to be compatible with various polymer substrates, and polyimide (PI) film was selected as a representative polymer substrate. In the laser scanning process, the laser beam was passed through the donor glass substrate vertically and was focused on the Ag NP layer. The focused laser beam was then scanned at the Ag NP layer, which was in contact with the polymer film underneath (Figure 1b). After the laser scanning process was finished, the donor glass substrate with the Ag NP was then detached from the acceptor polymer film (Figure 1c), leaving the polymer film with the selectively transferred Ag electrode (Figure 1d).

### 2.2. Optical Setup

The optical setup contained a 532 nm wavelength CW diode-pumped solid-state (DPSS) laser (Sprout-G-5W, Lighthouse Photonics, San Jose, CA, USA) which used Nd:YVO_4_ as its gain medium, while its spatial mode (Gaussian mode) was TEM_00_ at *M*^2^ = 1.0–1.1, with a beam diameter of 2.3 mm ± 10%. In the current study, the laser was focused with a 5× objective lens (M Plan Apo 5×, Mitutoyo, Kawasaki, Japan), and the sample was scanned using a motorized 2-axis translational stage (ANT130-060-XY-25DU-XY-CMS-MP-PLUS, Aerotech, Pittsburgh, PA, USA) along the programmed scanning path. For the laser scanning of the current study, the laser’s power was controlled in the ranges of 0.01–0.50 W and the scanning speed was fixed at 5 mm/s.

### 2.3. Measurements

The microscope images were taken with an optical microscope (BX53M, Olympus, Tokyo, Japan). Atomic force microscopy (AFM) and field emission scanning electron microscopy (FE-SEM), together with energy-dispersive X-ray spectroscopy (EDS) analysis, were conducted using an XE-100 from Park Systems and a MIRA3 from TESCAN (Brno, Czech Republic), respectively.

## 3. Results and Discussion

The proposed method shares common characteristics with the conventional SLS method [9,10], as the laser is utilized to convert the metal NP ink into a continuous conductive layer in a selective manner. At the same time, however, the objective of the current technique is to transfer the resultant conductive layer to the target polymer substrate and the current study should be classified as a laser transfer technique. It is noticeable that the proposed method possesses two differences compared to the conventional LIFT process. First, a pulsed laser is substituted by a CW laser that is more widely available. Secondly, the donor and the acceptor are in direct contact, while the gap distance between the two substrates had to be precisely controlled in the previous studies [17,18]. The experimental configuration is analogous to the shear-assisted laser transfer reported by our group previously [15], however, unlike the thin elastomer substrate, an arbitrary polymer film is not conformally attached to the metal NP layer. As a result, an additional glass substrate was added so that the target polymer substrate and the metal NP layer were sandwiched between two auxiliary glass substrates and directly touching each other.

**Figure 2:**
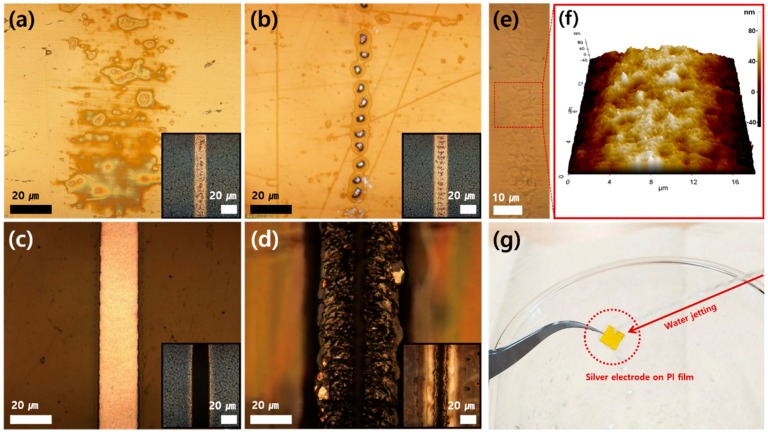
Parametric studies seeking the optimum laser parameters. Optical microscope images of the acceptor according to the laser power (laser velocity was fixed at 5 mm/s); (**a**) 210 mW, (**b**) 240 mW (**c**) 270 mW and (**d**) 400 mW (inset: corresponding donor substrate). (**e**) Optical and (**f**) AFM images of the PI film after etching the transferred Ag electrode. (**g**) Adhesion test against water-jetting.

The transfer characteristics are summarized in Figure 2. Upon successful transfer, we can observe the complementary results between the donor and the acceptor, i.e., a selectively sintered Ag electrode is created on the acceptor PI substrate, while the specific area of the laser-scanned Ag NP layer is removed from the donor glass substrate. Since the amount of the laser-induced heat is the most critical parameter for the features of the resultant, a parametric study was conducted by changing the laser power, while other variables such as the laser wavelength, the magnification of the objective lens and the spin coating condition were fixed. According to the laser power, we classified the transfer feature into four modes (Figure 2a–d). When the laser power is under 200 mW, it is noticeable that the Ag NP is sintered on the donor glass substrate, but there is no notable change on the acceptor PI film. By increasing the laser power to 200–230 mW, irregular patterns appear on the acceptor, while porous holes occur at the sintered Ag electrode on the donor (Figure 2a). We expect that the trapped solvent from the Ag NP ink layer is evaporated by the laser heating and condensed onto the acceptor PI film [17,29], leaving porous holes that originated from the rapid evaporation. Between 230 and 250 mW, regular Ag microparticles are transferred onto the acceptor PI film, while the sintered Ag electrode has complementary micro holes on the surface (Figure 2b). This phenomenon appears to be a typical balling effect [30,31] that is frequently reported in the relevant studies. The formation procedure, relevant factors that affect the results and the mechanism are very complex in the laser-induced balling effect, and since this is often considered as a defect in the typical SLS process, we attempted to find the optimum laser power that suppresses the balling phenomenon, which was experimentally found at 270 mW in this study (Figure 2c). At the optimum condition, a high-quality electrode is created at the acceptor while the Ag NP layer is sharply detached from the donor substrate. When the laser power overpasses 400 mW, the acceptor PI film is pyrolyzed and carbonized [32,33] (Figure 2d) to create a highly uneven structure.

Besides the successful demonstrations above, the stable and smooth transfer of the Ag electrode from the glass to the polymer substrate appears to require a supplementary explanation for its mechanism. We noted the asymmetric adhesion between the electrode and each substrate, and expected to find a reasonable explanation. To further discuss, firstly the adhesion mechanism between the PI substrate and the Ag electrode was investigated. Since it is reasonable to expect the adhesion force originates from the electrode-substrate interface, an investigation of the PI-electrode interface followed. To expose the interface, the Ag electrodes on the PI substrate were removed by chemical etching with a dilute HCl solution. Since PI is well-known for its excellent chemical stability, the removal process could preserve the surface morphology of the interface even after the etching process. Consequently, as shown in Figure 2e, we can identify the irregularities at the position of the removed electrode, which were further revealed as irregular dents and peaks of several micrometers by an AFM measurement (Figure 2f). Considering the intensive laser-thermal energy of focused laser irradiation, the rough surface morphology beneath the electrode could be ascribed to the thermal-deformation of the PI surface driven by the laser irradiation and the resulting thermal energy. Then, such rough topography of the PI surface could provide a plausible explanation for the adhesion at the interface in two ways, as follows. First, from a microscopic point of view, the increased interface area due to the rough topography can greatly enhance the molecular attractive interaction between the PI surface and the Ag electrode. Second, from a macroscopic point of view, the micrometer-sized dents and peaks could induce the mechanical interlocking of each side of the interface. As a result, the adhesion between the PI substrate and the Ag electrode seems to be attributable to the thermal-deformation of the PI substrate surface. On the other hand, the glass, the other side of the Ag electrode, is known to be far more stable to thermal-deformation. The melting point of glass reaches approximately 1500 °C, which exceeds not only the glass transition point of most polymers (~100 °C) but also the authors’ expectation of the laser-induced temperature with the power and scanning values of this study. Therefore, in this case, we could not expect the adhesion mechanism described above, unlike in the case of the PI-electrode interface. Consequently, the above-described thermal deformation induced adhesion mechanism provides a possible explanation for the asymmetric adhesion between the electrode and each substrate. The difference in thermal stability between the PI and the glass substrates generates the asymmetric adhesion that can result in the transfer phenomenon. In addition to the effect of the surface morphology, it is well known that the noble metals do not adhere well to glass [34,35], while the enhanced adhesion between metal and polymer substrate after the SLS process has been experimentally confirmed by numerous studies [9,10]. Meanwhile, enhanced surface adhesion by photothermal reactions has been studied as the driving force of the laser-induced thermal imaging (LITI) method as well [36]. The adhesion of the Ag electrode after the transfer was further tested by jetting water using a squeeze bottle with a nozzle (Figure 2g). It was confirmed that vigorous cleaning using the wash bottle does not result in detachment of the resultant electrode.

**Figure 3:**
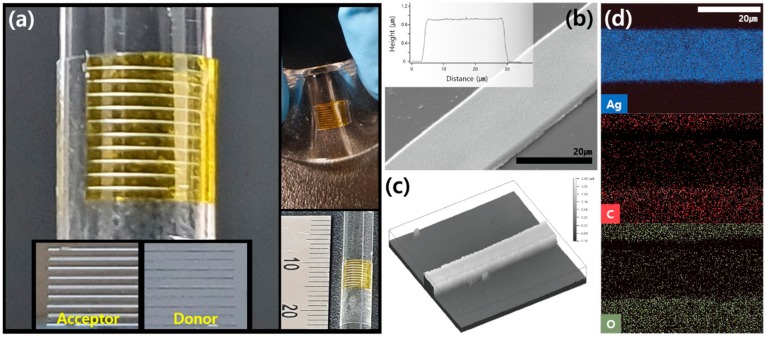
(**a**) Photographs of the transferred Ag electrodes on non-flat substrates such as a cylindrical rod and a bent elastomer (inset: photograph of the acceptor and the donor together with a scale to check the size). (**b**) SEM (inset: AFM), (**c**) optical surface profiler and (**d**) EDS measurements of the transferred Ag electrode.

Through this proposed method, we could create micro Ag electrodes on the flexible PI film that can be attached to arbitrary irregular shapes such as a cylindrical rod (Figure 3a). For more accurate observation of the created Ag electrode, we measured the transferred Ag electrode with SEM, AFM, an optical surface profiler and EDS (Figure 3b–d). The SEM (Figure 3b) and optical surface profiler (Figure 3c) images show that the selective transfer was achieved on the PI film and also that the transferred Ag electrode has a uniform surface composition. We expect that the simultaneous sintering and transfer processes by the rapid photothermal reaction, together with extremely high temperature gradient, create the Ag electrode without any excessive substances. The AFM measurement (Figure 3b inset) verifies the uniform and flat-top surface of the Ag electrode, and the EDS image (Figure 3d) confirms that the transferred electrode is indeed derived from the Ag NP.

**Figure 4:**
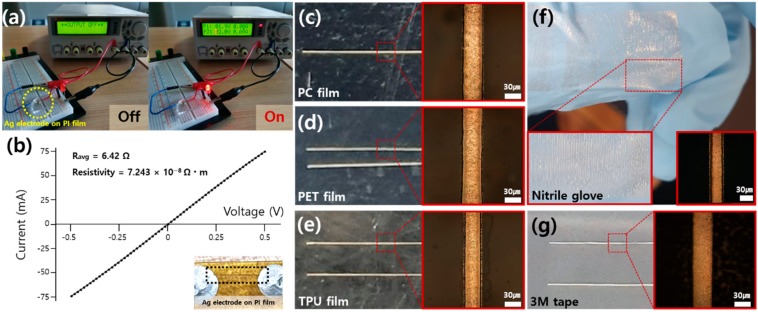
(**a**) Photographs of the circuit using the transferred Ag electrode on the PI film. (**b**) I-V characteristics of the transferred Ag electrode. Ag electrode transferred to (**c**) PC film, (**d**) PET film, (**e**) TPU film, (**f**) Nitrile glove and (**g**) 3M tape. The insets of (**c**–**g**) are the optical microscope images of the corresponding Ag electrodes.

To ensure the electrical conductivity of the resultant, the transferred Ag electrode was connected to power a light-emitting diode (LED). The photographs (Figure 4a) indicate that the Ag electrodes created by the proposed method are available as efficient conductors. From the resistance and the dimensions of the transferred Ag electrode, we obtained a resistivity of 7.243 × 10^−8^ Ω·m for the current conductor, which is about five times higher than that of bulk silver (1.59 × 10^−8^ Ω·m). This process is directly applicable to other flexible substrates (Figure 4e–g) as shown in the representative examples of the transferred Ag electrode on the polycarbonate (PC) film (Figure 4c), polyethylene terephthalate (PET) film (Figure 4d), thermoplastic polyurethane (TPU) film (Figure 4e), and even on a commercial nitrile glove (Figure 4f) and 3M tape (Figure 4g). For these substrates, the transfer of the Ag electrode occurs effectively at a similar laser power, and we expect that the transferred mechanism is analogous in every case.

As shown in Table 1, the laser-induced transfer method has been mainly studied in the LIFT method using a pulsed laser, which inevitably involves additional heat treatment. There have been several attempts to improve this conventional method. P. Sopeña et al. [23] presented LIFT using a CW laser and secured its possibility as a low-cost fabrication method, but this scheme also requires additional heat treatment because it does not include a simultaneous sintering process. Shin et al. [15] introduced the shear-assisted laser transfer method using a CW laser, which utilizes the thermal expansion coefficient differences between the donor glass substrate and the acceptor Polydimethylsiloxane (PDMS) in conformal contact. However, the shear-assisted laser transfer method requires a substrate with high transparency and thermal expansion coefficients, making it difficult to apply to arbitrary polymer substrates.

## 4. Conclusions

The laser-induced transfer method of metal NPs using a CW laser, apart from the conventional LIFT method using a pulsed laser, has hardly been developed to date, since the main transfer mechanism has remained as jet formation. The current study reports the micropatterning of the target metal NP onto a flexible acceptor substrate by harnessing the different adhesion strengths that originate from the thermal deformation. Through the proposed process, the sintering and transfer occur simultaneously at a single optical platform without the need for the additional cleaning process. As a result, the proposed method could be an efficient integrated and complementary technique for the previous SLS and LIFT process for metal NPs. We expect that the current study can be applied to a wide range of substrates with enough heat resistance to endure the sintering temperature of the metal NPs. Furthermore, we anticipate that this method is principally material-independent, such that an identical process would be compatible with a variety of metal NPs. The proposed CW laser-induced transfer method, therefore, appears to possess a large potential as a facile manufacturing scheme for flexible electronics.

## Figures and Tables

**Table 1:** Recent progress in the laser-induced transfer of metal NPs.

	Laser	Laser Condition	Donor	Acceptor	Gap distance	Year	Material	Method	Ref.
1	Pulsed laser (ns)	266 nm 40–100 mJ/cm^2^	Quartz	SiO_2_	300 µm	2014	Silver	LIFT Additional Heat treatment	[37]
2	Pulsed laser (fs)	1027 nm 530 mJ/cm^2^	Glass	Glass	200 µm	2015	Silver	LIFT Additional Heat treatment	[21]
3	Pulsed laser (fs)	1027 nm 530 mJ/cm^2^	Glass	Glass with ablated channel	160 µm	2016	Silver	LIFT Additional Heat treatment	[20]
4	Pulsed laser (ps)	343 nm 35–64 mJ/cm^2^	Quartz	Glass	230 µm	2016	Silver	LIFT Additional Heat treatment	[19]
5	Pulsed laser (ns)	1064 nm 2–6 J/cm^2^	Glass	Glass	150 µm	2017	Silver	LIFT	[38]
6	Continuous wave laser	1064 nm 1W 600mm/s	Glass	Glass	150 µm	2017	Silver	CW-LIFT Additional Heat treatment	[23]
7	Pulsed laser (ns)	1064 nm 11000 mJ/cm^2^	Glass	Paper	150 µm	2018	Silver	LIFT Additional Heat treatment	[22]
8	Continuous wave laser	532 nm 0.54 W 140 mm/s	Glass	PDMS	Contact	2018	Silver	Shear-assisted	[15]
9	Pulsed laser (ns)	532 nm 320 mJ/cm^2^	Glass	Glass SU-8 film Organic layer	150 µm 500 µm 3000 µm	2019	Silver	LIFT Additional Heat treatment	[39]
10	Pulsed laser (ns)	532 nm 360 mJ/cm^2^	Quartz	SU8 on glass	100 µm	2019	Copper	LIFT Post sintering	[40]
11	Pulsed laser (ps)	532 nm 80–240 nJ (burst mode)	Glass	Glass	200 µm	2019	Silver	LIFT(burst)	[41]
Current Study	Continuous wave laser	532 nm 0.27 W 5 mm/s	Glass	Polymer Substrates	Contact		Silver	Enhanced adhesion by CW laser	
